# Clinical evolution of mediastinitis in patients undergoing adjuvant hyperbaric oxygen therapy after coronary artery bypass surgery

**DOI:** 10.1590/S1679-45082013000300014

**Published:** 2013

**Authors:** Julyana Galvão Tabosa do Egito, Cely Saad Abboud, Aline Pâmela Vieira de Oliveira, Carlos Alberto Gonçalves Máximo, Carolina Moreira Montenegro, Vivian Lerner Amato, Roberto Bammann, Pedro Silvio Farsky

**Affiliations:** 1 Instituto Dante Pazzanese de Cardiologia, São Paulo, SP, Brazil

**Keywords:** Hyperbaric oxygenation, Mediastinitis/therapy, Myocardial revascularization, Osteomyelitis, Surgical wound infection/therapy

## Abstract

**Objective:**

To evaluate the use of hyperbaric oxygen therapy as an adjunctive treatment in mediastinitis after coronary artery bypass surgery.

**Methods:**

This is a retrospective descriptive study, performed between October 2010 and February 2012. Hyperbaric oxygen therapy was indicated in difficult clinical management cases despite antibiotic therapy.

**Results:**

We identified 18 patients with mediastinitis during the study period. Thirty three microorganisms were isolated, and polymicrobial infection was present in 11 cases. *Enterobacteriaceae* were the most prevalent pathogens and six were multi-resistant agents. There was only 1 hospital death, 7 months after the oxygen therapy caused by sepsis, unrelated to hyperbaric oxygen therapy. This treatment was well-tolerated.

**Conclusion:**

The initial data showed favorable clinical outcomes.

## RESUMO

**Objetivo:**

Avaliação da utilização de oxigenoterapia hiperbárica, como tratamento adjuvante, em casos de mediastinite, em pós-operatório de cirurgia de revascularização miocárdica.

**Métodos:**

Estudo descritivo retrospectivo, no período entre outubro de 2010 e fevereiro de 2012. A oxigenoterapia hiperbárica foi indicada nos casos de difícil manejo clínico a despeito da antibioticoterapia.

**Resultados:**

Identificaram-se 18 pacientes com mediastinite, nos quais 33 microrganismos foram isolados, estando a infecção polimicrobiana presente em 11 casos. Enterobactérias foram os germes mais prevalentes e seis agentes multirresistentes. Ocorreu 1 óbito, na evolução, 7 meses após o término da oxigenoterapia, por septicemia, não relacionado à terapêutica. O tratamento foi bem tolerado.

**Conclusão:**

Os resultados clínicos iniciais foram favoráveis.

## INTRODUCTION

Deep wound infection after surgery is a severe complication of coronary artery bypass surgery (CABS). It is responsible for high mortality and morbidity rates, significant increase in hospital and medical costs and longer hospital stay. Deep wound infection incidence may range between 0.4% and 5.3%, and its inhospital mortality ranges from 10 to 47%. In the institution where the study was conducted mediastinitis incidence is 0.5% to 1% and the mortality rate is 8.4%^(1–5)^.

Poststernotomy deep infection treatment remains a challenge. The advances achieved up to the present days provided new therapeutic options for these severe infections. Evidences suggest that aggressive and early approaches associated with the use of antimicrobials constitute an important treatment option^(6,7)^.

In this context, hyperbaric oxygen therapy (HBO) appears as an adjuvant therapy for deep surgical wounds treatment. This technique has been used since the 1930s and consists of 100% oxygen administration within an environmental pressure higher than the atmospheric pressure at sea level using specific chambers which increase arterial oxygen content at up to 20 times^(3,8)^.

HBO treatment provides favorable biochemical and cellular effects to surgical wounds. Among these effects are reversal of tissue hypoxia, increased phagocytic ability toward some bacteria, and stimulus on collagen matrix formation. These effects are vital to angiogenesis and tissue healing, and as a result, to the improvement of microvascular perfusion ^(3,6,8)^.

The use of HBO is suggested as treatment option to non-healing surgical wounds secondary to inflammatory process by the European Committee for Hyperbaric Medicine (ECHM)^(9).^ Evidences on use of HBO as an adjuvant therapy for deep wound infections after CABS are scarce and further studies are needed to elucidate its benefits.

## OBJECTIVE

To evaluate the use of HBO as an adjuvant therapy in 18 mediastinitis cases in the postoperative CABS.

## METHODS

*Instituto Dante Pazzanese de Cardiologia*, located in São Paulo - Brazil, is a public teaching hospital with 350 beds for cardiovascular surgery. Roughly 2,000 heart surgeries are performed annually, and of this total about 1,000 are CABSs. Our facility has an infection control and prevention program, and also performs active disease surveillance following the Center for Disease Control and Prevention (CDC) criteria^(10)^.

This study was approved by the Institutional Ethical Committee, protocol number 4,293.

### Study design

This is a descriptive and retrospective study carried out from October 2010 to February 2012. The study population was composed by mediastinitis cases reported according to CDC criteria^(10)^. In the study period the mediastinitis incidence at our institution was 1% (23/2,241 heart surgeries).

HBO was indicated in cases with surgical wound discharge, an extensive bleeding area and a hard-to-heal wound despite antibiotic therapy. We evaluated clinical data from medical records and post-therapeutic evolution from 30 days follow-up to 1 year after hospital discharge.

To identify microorganisms in the surgical wound we took swab culture samples and for the antibiogram we used VITEK^TM^ 2. For *Enterobacteriaceae* and *Pseudomonas aeruginosa* the sensitivity profile was confirmed using the disk-diffusion test. To methicillin-resistant *Staphylococcus* the minimal inhibitory concentration (MIC) was obtained by VITEK^TM^ 2 and confirmed by the e-Test^TM^.

All patients received initial empiric antibiotic therapy in accordance with the institutional protocol, which later was adjusted based on sensitivity profile of the isolated microorganism.

### Hyperbaric oxygen therapy

After signing the consent form the patients were treated with HBO using a monopatient equipment with constant flow of 100% oxygen and with deep time of 90 minutes (pressure, time of treatment). Of the 18 patients, 13 were treated at 2.5 atmospheres absolute (ATA) and 3 were initially treated at 2.5 ATA in the first 3 sessions and at 2.0 ATA in the other sessions. For clinical reasons the pressure was reduced, which was justified for renal impairment and/or lower ejection fraction (≤30%) during hospital stay.

Treatment sessions were conducted daily for five days a week.

## RESULTS

We selected 18 patients to undergo HBO; on clinical examination these patients presented discharge from the surgical wound, an extensive bleeding area and a hard-to-heal wound.

Patients underwent around 11.5 treatment sessions ranging from 5 to 20 sessions. The criterion to stop HBO was clinical improvement in the surgical wound initial appearance.

Two patients refused to participate for claustrophobia and were excluded. We did not observe any other events or side effects during HBO sessions.

Clinical and demographic characteristics of participants are described on table 1. It is important to mention the high prevalence of women, body mass index (BMI) >30kg/m^2^ in 11 patients, and BMI >40kg/m^2^ in 3 individuals. In addition, a high prevalence of *diabetes mellitus* among patients was observed. Seven patients had moderate to severe left ventricular dysfunction.

**Table 1:** Clinical and demographic characteristics of patients submitted to hyperbaric oxygenotherapy

Characteristics		n (%)
Age (years)		61.6±10.5
Women		13 (72)
BMI (kg/m^2^)		
	<30	7 (38)
	30-40	8 (44)
	>40	3 (17)
Dyslipidemia		15 (83)
Diabetes mellitus		12 (67)
SH		18 (94.4)
Smoking		5 (28)
Previous AMI		9 (50)
Cerebrovascular disease		2 (11)
Peripheral artery disease		3 (17)
COPD		2 (11)
Ventriculography		
	Normal	8 (44)
	Mild	3 (17)
	Moderate	5 (27.7)
	Severe	2 (11)
Creatinine clearance		77.4±31.5
Total		18 (100)

BMI: body mass index; SH: systemic hypertension; AMI: acute myocardial infarction; COPD: Chronic Obstructive Pulmonary Disease.

Infection risk was calculated using the Society of Thoracic Surgery (STS) score^(11)^ (Table 2). Only two patients had low score for perioperative infection risk (<7).

**Table 2:** Estimated infection risk based on score of the Society of Thoracic Surgery score

Patient	Preoperative score	Infection risk (%)	Combined	Infection risk (%)
1	10	3.1	9	2.7
2	22	11.4	20	10.2
3	12	4.0	9	2.7
4	12	4.0	11	3.5
5	17	7.4	15	6.0
6	15	5.8	14	5.2
7	18	8.2	22	11.8
8	17	7.4	15	6.0
9	18	8.2	19	9.4
10	8	2.4	6	1.8
11	17	7.4	20	10.2
12	4	1.5	3	1.2
13	15	5.8	14	5.2
14	29	9.9	17	7.6
15	6	1.8	6	1.8
16	8	2.4	6	1.8
17	8	2.4	9	2.7
18	17	7.4	14	5.2

After analyses of intraoperative data we observed that the number of grafts used was 2.7±0.6 and no cases of double mammary graft. The extracorporeal circulation (ECC) mean time was 87.2±23.7 minutes.

A total of 33 microorganisms were isolated in 18 patients being the polymicrobial infection found in 11 cases. The microorganisms isolated in surgical wound are described on table 3. Of them, six were multiresistance considering different types of antimicrobials.

**Table 3:** Distribution of microorganisms that caused infection

Microorganisms	n (%)
*Enterobacteriaceae*	10 (30.0)
Coagulase-negative staphylococci	7 (21.0)
Ertapenem-resistant *Klebsiella pneumoniae*	4 (12.0)
Hodge+*	3
Hodge-	1
Extended spectrum β-lactamase	3 (9.0)
Oxacillin-sensible *Staphylococcus aureus*	2 (6.0)
*Enterococcus spp*	2 (6.0)
*Pseudomonas aeruginosa*	2 (6.0)
*Acinetobacter baumanni*	1 (3.0)
*Candida tropicalis*	1 (3.0)
Oxacillin-resistant *Staphylococcus aureus*	1 (3.0)

* In this group of patients two bacteria were identified as *Klebsiella pneumoniae carbapenemase* (KPC) using the polymerase chain reaction.

**Figure 1:**
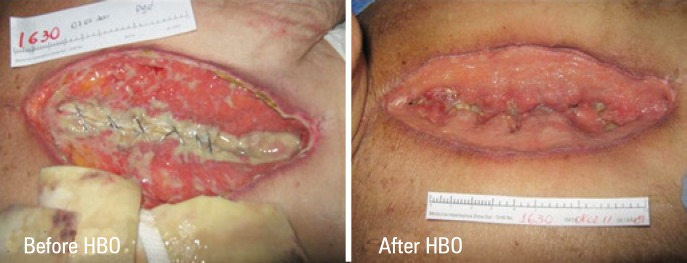
Clinical evolution of surgical wound after nine HBO sessions associated with antibiotic therapy

On table 4, we described the clinical evolution of patients submitted to HBO. Figure 1 presents the clinical evolution of one patient included in the study.

**Table 4:** Clinical evolution of patients after hyperbaric oxygen as an adjuvant therapy

Characteristics		Data
Antibiotic therapy (mean/days)		46±15.6
Hospital stay (mean/days)		68±41.2
Hyperbaric oxygenotherapy (mean/sessions)		11.4±3.6
Complications during hospital stay, n (%)		
	After resuture dehiscence	6 (37.5)
	Death*	1 (6.3)
	Osteomyelitis	3 (18.8)
Complication after discharge (1 year), n (%)		
	After resuture dehiscence	0
	Osteomyelitis	1 (6.3)
	Late death (1 year)	0

* Death occurred due to sepsis 7 months after treatment for clinical complications unrelated to HBO.

## DISCUSSION

The main and most fearsome infectious complications post-sternotomy are mediastinitis and osteomyelitis, which are reported to occur in up to 4% of patients^(11)^.

This study sought to verify HBO as an adjunctive therapy for the treatment of deep infections after sternotomy. It was carried out because of the difficulties in clinical management of some mediastinitis patients and lack of evidences in the literature on this specific subject^(12,13)^.

At our institution mediastinitis observed in the study period was 1% of the cardiac surgery cases. Demographic characteristics and risk factors for this infection were already well-described in the literature and at our institution^(4,11,14)^.

The benefits of using HBO are described in a number of clinical and surgical conditions with promising results^(15,16)^ by reducing length of hospital stay and hospital costs^(12)^.

An important clinical improvement was seen in the study population by the reduction of discharge from the wound and in bleeding areas, enabling a surgical approach for possible resuture. Barili et al.^(17)^ and Strecker et al.^(18)^ had already described HBO benefits for deep sternal infection.

Two patients did not undergo the treatment due to claustrophobia, which was above the mean. We attributed that for the patient's own illness awareness facing a long hospital stay as a cause of resistance to treatment. These two patients were excluded from the analysis.

Six multi-resistant agents were seen, three were identified as *Klebsiella pneumonia* according to the positive modified Hodge test, and the other two cases (one *Acinetobacter baumannii* and another *Pseudomonas aeruginosa*) were confirmed by polymerase chain reaction (PCR).

All coagulase-negative staphylococci were oxacillin-resistant, in addition a methicillin-resistant *Staphylococcus aureus* (MRSA) was found. The polymicrobial infections (61%) could be explained by the long hospital stay with subsequent patient colonization and a broken skin barrier. The swab culture collection may also justify this fact.

Different from previous studies^(13,14–20)^, we observed a higher prevalence of gram-negative agents (60.6%) whereas gram-positive represented 36.3% of cases. However, in our service, we did not find differences between the infection risk score for gram-positive and gram-negative agents^(13)^.

Even after HBO use as an adjuvant therapy along with antibiotic therapy we found, during the patients hospital stay, dehiscence after resuture in six cases (37.5%) and osteomyelitis in three cases (18.8%). We believe that such fact occurred because of the patients' vasculopathy. One patient died after 7 months of CABS for other complications unrelated to HBO.

### Study limitations

This descriptive and retrospective study was carried out at a single center without a control group, therefore, it has limitations related to its methodology. However, because this is an initial study with high number of cases on HBO adjuvant treatment for mediastinitis after CABS, it brings new perspectives and generates hypothesis that should be confirmed in further prospective and randomized studies.

## CONCLUSION

HBO as an adjunctive therapy for treatment of mediastinis patients after CABS had favorable clinical results in this study population.
